# Metabolic-inflammatory heterogeneity in PCOS assisted reproduction: a compartmental susceptibility model

**DOI:** 10.3389/fendo.2026.1963493

**Published:** 2026-10-09

**Authors:** Lin Yuying

**Affiliations:** Department of Academic Affairs, First Affiliated Hospital of Heilongjiang University of Chinese Medicine, Harbin, China

**Keywords:** assisted reproductive technology, endometrial receptivity, follicular microenvironment, metabolic inflammation, polycystic ovary syndrome, polyendocrine metabolic ovarian syndrome, precision medicine

## Abstract

Polycystic ovary syndrome (PCOS) is a heterogeneous endocrine-metabolic disorder rather than a uniform reproductive diagnosis. Women entering assisted reproductive technology (ART) differ in adiposity, insulin sensitivity, lipid metabolism, androgen excess, inflammatory activity, and oxidative stress, but these systemic traits do not map uniformly onto the follicular or endometrial compartments. We propose a testable compartmental susceptibility model in which systemic metabolic-inflammatory burden acts as a background exposure and clinically relevant effects emerge when stage-specific follicular-oocyte or endometrial-maternal vulnerability is also present. Evidence was interpreted with a pragmatic hierarchy that separates guideline/RCT evidence, registry and externally validated prognostic evidence, adjusted observational associations, mechanistic discovery studies, and protocol-only evidence. Current data support metabolic assessment, prevention of excessive ovarian response, and maternal risk surveillance, but they do not validate a metabolic-inflammatory subtype for selecting stimulation, trigger, frozen-embryo-transfer preparation, immune adjuncts, or other phenotype-specific treatment. Local immune, extracellular-vesicle, and multi-omics signals remain discovery tools rather than clinical tests. Precision ART in PCOS will require paired systemic-local phenotyping, external validation, and prospective phenotype-by-treatment interaction studies using cumulative live birth and maternal-neonatal safety as primary endpoints.

## 1 Introduction

In 2026, an international consensus adopted polyendocrine metabolic ovarian syndrome (PMOS) as the new name for polycystic ovary syndrome (PCOS), emphasizing its multisystem endocrine and metabolic biology rather than an ovarian-cyst label alone (1). Because nearly all ART studies reviewed here enrolled populations under the term PCOS, we use PCOS throughout the manuscript for consistency with the source literature. PMOS is mentioned only here as the updated nomenclature. The 2023 international evidence-based guideline likewise emphasizes heterogeneous reproductive and metabolic manifestations and integrated assessment across the life course (2).

For assisted reproduction, this heterogeneity has direct clinical relevance. Insulin resistance, adiposity, dyslipidemia, hyperandrogenism, oxidative stress, and chronic low-grade inflammation can coexist in different combinations and at markedly different levels of severity. These features may be associated with ovarian response, oocyte and embryo development, endometrial preparation, ovarian hyperstimulation syndrome (OHSS), pregnancy maintenance, and maternal-neonatal outcomes. Importantly, the association depends on the endpoint: a metabolic trait may relate differently to laboratory outcomes and to cumulative live birth (3, 4).

Recent reviews have already summarized the broad pathophysiology and treatment landscape of PCOS, including insulin resistance, inflammation, oxidative stress, genetic susceptibility, and microbiome-related mechanisms (5, 6). Their contribution is primarily disease-level synthesis. The unresolved ART question is narrower: whether a systemic phenotype is informative at a specific treatment stage, whether a local follicular or endometrial measurement adds information beyond routine clinical variables, and whether any observed association actually changes a treatment decision. This distinction defines the scope of the present review and avoids presenting familiar PCOS mechanisms as novel in themselves.

Single-variable approaches can obscure this complexity. Body mass index (BMI), a binary definition of insulin resistance, or one circulating inflammatory marker is easy to measure, but these variables are not interchangeable and may be biologically distant from the reproductive compartment of interest. In PCOS IVF/ICSI cohorts, adiposity, glucose and lipid metabolism, and inflammatory indices have shown interdependent associations with oocyte and embryo outcomes. The mediation analyses often cited in this context are statistical decompositions of observational data, not demonstrations of a biological causal pathway; their estimates depend on strong assumptions about measured and unmeasured confounding, exposure-mediator relations, and model specification (7, 8). Local studies identify granulosa-cell, follicular-fluid, extracellular-vesicle, and immune signals that cannot be inferred directly from BMI, but these findings are predominantly mechanistic or exploratory (9–11).

After embryo creation, the same need for stage-specific interpretation remains. Higher triglyceride-glucose body mass index (TyG-BMI) has been associated with miscarriage, live birth, and selected obstetric outcomes after frozen embryo transfer (FET) in women with PCOS (12), while meta-analysis links PCOS in ART pregnancies with several maternal and neonatal complications (13). These findings are compatible with two partially separable routes—a follicular-oocyte pathway and an endometrial-maternal pathway—but they do not establish that either route is causal in an individual patient.

This review therefore uses a three-dimensional framework—phenotype domain × sampling compartment × outcome/decision stage—to organize the evidence rather than to create a diagnostic subtype system. We prioritize peer-reviewed human ART cohorts, interventional studies, large registries, systematic reviews and guidelines, and mechanistic studies that directly address follicular, endometrial, or clinical outcomes. A targeted update through September 2026 was used to incorporate recent evidence. To avoid flattening very different study designs into a single evidentiary level, we apply the pragmatic hierarchy summarized in **Table 1**: treatment-effect claims require randomized or guideline-level evidence; registry and cohort data support prognosis or benchmarking; small tissue and multi-omics studies support biological plausibility; and trial protocols are not used as evidence of efficacy.

**Table 1:** Pragmatic evidence hierarchy, principal bias domains, and allowable inference.

Tier	Typical evidence	What it can support in this review	Principal bias/uncertainty	Clinical interpretation
A	Evidence-based guideline; systematic review/meta-analysis of RCTs; adequately conducted RCT	Treatment-effect inference within the studied population and protocol	Risk of bias, imprecision, heterogeneity, indirectness	Highest weight for management; still protocol- and outcome-specific
B	Large registry; multicenter prospective cohort; externally validated prognostic model	Benchmarking, prognosis, transportability, external validation	Residual confounding, variable definitions, missingness, treatment selection	Supports risk estimation, not treatment causality
C	Adjusted observational cohort, often retrospective or single-center	Association and hypothesis generation	Selection bias; confounding by age, reserve, BMI/weight change, stimulation/transfer strategy and laboratory practice	Does not establish mediation, mechanism, or treatment benefit
D	Small mechanistic, tissue, immune, proteomic, metabolomic or other omics study	Biological plausibility and candidate-marker discovery	Small samples, late/invasive sampling, batch effects, multiplicity, overfitting, limited replication	Research-stage only; not a routine clinical test
P	Trial registration/protocol without outcome data; preclinical-only evidence	Research agenda only	No clinical outcome evidence	Not used to support efficacy or clinical recommendations

This hierarchy is a pragmatic weighting scheme for a narrative review, not a formal GRADE assessment. Study-specific certainty may be lower than the tier implied by design alone; conclusions were limited to the allowable inference shown above.

To add a testable conceptual layer, we propose a compartmental susceptibility hypothesis. Systemic metabolic-inflammatory burden is treated as a background exposure, whereas the follicular and endometrial compartments differ in susceptibility or buffering capacity. The model generates three falsifiable predictions: first, paired systemic-local measurements or their discordance should predict stage-specific outcomes better than a systemic marker alone; second, follicular signals should add most information for oocyte/embryo endpoints whereas endometrial-maternal signals should add most information after transfer; and third, a phenotype becomes clinically actionable only when a prespecified phenotype-by-treatment interaction shows that management changes improve patient-important outcomes. **Figure 1** depicts this model as a research framework, not a treatment algorithm.

**Figure 1:**
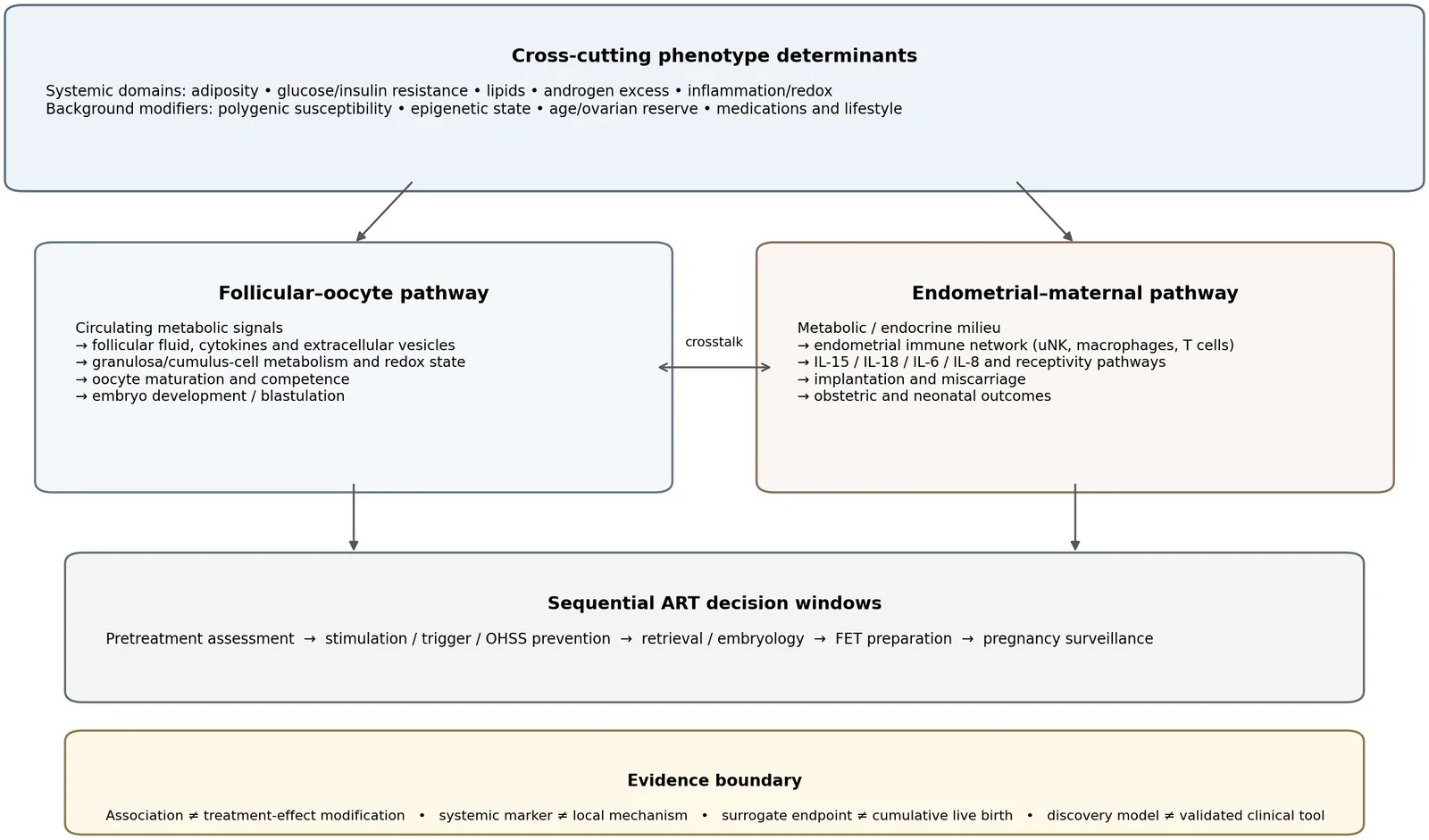
Compartmental susceptibility model linking systemic metabolic-inflammatory burden with stage-specific follicular-oocyte and endometrial-maternal vulnerability across ART. The testable proposition is that paired systemic-local information and phenotype-by-treatment interactions will be more informative than a single systemic marker. The model is hypothesis-generating and is not a validated subtype or treatment algorithm. uNK, uterine natural killer; OHSS, ovarian hyperstimulation syndrome; FET, frozen embryo transfer.

## 2 ART-relevant metabolic-inflammatory phenotypes

### 2.1 Phenotype domains, compartments, and outcomes

An ART-relevant phenotype must specify what is measured, where it is measured, and which decision or outcome the measurement could plausibly inform. The same patient may have discordant systemic, follicular, and endometrial signals, and the same biomarker can have different implications for ovarian response, embryo competence, transfer outcome, and obstetric risk. Treating PCOS as one uniform exposure therefore risks mixing distinct biological and clinical questions.

For this purpose, three dimensions are useful: phenotype domain (adiposity, glucose-insulin metabolism, lipids, androgen excess, inflammation, and oxidative stress); measurement compartment (anthropometric/circulating, follicular-fluid/granulosa, oocyte/omics, and endometrial/maternal); and outcome layer (ovarian response and OHSS, oocyte and embryo development, implantation and pregnancy loss, cumulative live birth, and maternal-neonatal safety). Keeping these dimensions separate helps prevent a systemic marker from being treated as a local mechanism or a laboratory surrogate from being treated as patient benefit.

### 2.2 Systemic phenotype domains

Body mass index is the most frequently used indicator of adiposity in ART studies, but it is an incomplete phenotype descriptor. It does not distinguish fat distribution, lean mass, dyslipidemia, insulin resistance, or inflammatory activity, and identical BMI categories may contain metabolically different women. Comparisons of lean and obese PCOS cohorts generally support clinically relevant heterogeneity, including differences in cumulative live-birth patterns, but residual confounding by age, treatment strategy, metabolic comorbidity, and embryo-transfer practice remains difficult to eliminate (14, 15). BMI should therefore be treated as an entry variable rather than as a complete metabolic classification.

Lipid measures add information but also illustrate the danger of binary labeling. In a large PCOS cohort undergoing a first complete IVF/ICSI cycle, overall cumulative outcomes were similar between broadly defined dyslipidemia and control groups, whereas specific total-cholesterol levels showed a more adverse association with cumulative live birth (3). This pattern suggests that a composite dyslipidemia diagnosis may obscure the direction, magnitude, or threshold behavior of individual lipid components. Studies linking adiposity and lipid indicators with oocyte or embryo outcomes also report correlated changes in triglycerides, total cholesterol, low-density lipoprotein cholesterol, and high-density lipoprotein cholesterol, making it difficult to isolate one causal exposure (7, 8). For ART-oriented phenotyping, lipid values should therefore be recorded as continuous and component-specific measures whenever possible, alongside adiposity and glucose metabolism.

Insulin resistance is central to many metabolic interpretations of PCOS, but its operational definition varies across fasting indices, oral glucose tolerance test-derived measures, and study-specific cutoffs. These methods identify overlapping rather than identical populations. In IVF/ICSI cohorts, higher glucose- and insulin-related indices have been associated with oocyte yield, mature oocytes, embryo quality, or later reproductive outcomes, but the direction and persistence of associations depend on the index, adjustment set, and outcome (4, 8). A retrospective first-transfer study also associated increasing homeostatic model assessment of insulin resistance (HOMA-IR) strata with early miscarriage, live birth, and macrosomia (16). These are prognostic associations, not evidence that HOMA-IR itself is a causal treatment target.

These observations justify retaining insulin resistance as a major phenotype domain, but no single cutoff should be treated as universally applicable. Fasting measures may miss post-load abnormalities, oral glucose tolerance test-derived indices require additional sampling, and thresholds can shift with population characteristics and laboratory methods. Insulin-resistance status is therefore best represented by the exact assay and definition used, together with continuous glucose and insulin measures and the biological compartment or outcome under study.

Low-grade inflammation and oxidative stress are often invoked as bridges between adiposity, insulin resistance, ovarian dysfunction, and adverse ART outcomes. In PCOS IVF/ICSI cohorts, white blood cell count and related inflammatory measures have been associated with adiposity, lipids, oocyte development, and embryo development; mediation models suggest that inflammatory activity may partly account for some observed metabolic associations (7). However, mediation in observational data is sensitive to model assumptions and does not prove a biological causal chain. Inflammation should consequently be recorded as a phenotype component whose mechanistic role requires triangulation with local and experimental evidence.

Immune activity also differs by compartment. High-dimensional profiling has shown that follicular fluid and systemic circulation contain distinct immune-cell compositions and cytokine environments, with PCOS-related differences detectable within the follicular compartment (11). Thus, a normal or elevated circulating inflammatory marker cannot be assumed to describe the peri-oocyte immune milieu. Oxidative stress and androgen excess are similarly intertwined with metabolic state and ovarian function, but assays, timing, and adjustment strategies vary substantially. They are retained as explicit domains in the map while their independent contributions are treated as uncertain unless supported across measurement levels.

### 2.3 Local versus systemic measurement

To prevent conceptual slippage, phenotype domains must be separated by sampling compartment. Systemic measures include anthropometry, fasting or post-load glucose and insulin, lipid fractions, circulating androgens, and inflammatory or oxidative-stress markers. These variables are clinically accessible before treatment, but their ART associations may be confounded by age, ovarian reserve, infertility duration, prior treatment, weight change, stimulation protocol and gonadotropin exposure, trigger strategy, and coexisting endocrine or metabolic disease.

Follicular and cellular measures include follicular-fluid metabolites and cytokines, extracellular vesicles, granulosa-cell expression, immune-cell composition, and integrated transcriptomic, proteomic, or metabolomic signatures. Obesity and PCOS have been associated with differences in follicular-fluid extracellular-vesicle protein profiles, and multi-omics analyses have identified candidate pathways associated with fertilization or early embryo measures (10, 17). These studies increase biological resolution, but small samples, batch effects, multiple testing, and limited external replication place them in a discovery or mechanistic-evidence tier rather than a clinical-test tier.

Endometrial and maternal measures concern implantation conditions, transfer-cycle context, pregnancy maintenance, and obstetric or neonatal outcomes. TyG-BMI, insulin-resistance indices, and related metabolic measures have been associated with FET or pregnancy outcomes in observational PCOS cohorts (12, 16), while meta-analysis indicates associations between PCOS and several maternal or neonatal complications after ART (13). These results cannot distinguish endometrial, placental, systemic, treatment-selection, or residual-confounding pathways and should not be interpreted as proof of a local endometrial mechanism.

The practical implication is that evidence should be interpreted within the compartment in which it was measured. Systemic biomarkers are attractive for pretreatment assessment, whereas follicular and endometrial measurements may be more biologically specific but less accessible and often become available only after treatment has started. None of these measurement levels currently provides validated universal thresholds for treatment selection.

### 2.4 Connecting phenotype domains to ART outcome layers

#### 2.4.1 Ovarian response, oocyte competence, and embryo development

Systemic metabolism may influence early ART outcomes through correlated rather than isolated pathways. Analyses of PCOS IVF/ICSI cohorts suggest that glucose dysregulation, adiposity, lipids, and inflammation may jointly relate to the number or maturity of retrieved oocytes and embryo development (7, 8). Oral glucose tolerance test-based insulin-resistance analyses further indicate that embryo-quality associations can differ between women with and without PCOS (4). These findings support multivariable phenotyping, but they do not establish that correcting one marker will necessarily normalize the local follicular environment or improve live birth.

The local compartment provides a mechanistic bridge but also introduces additional heterogeneity. Granulosa-cell ANGPTL4 expression has been associated with PCOS and metabolic features (9), extracellular-vesicle proteomics vary with obesity and PCOS status (10), and follicular multi-omics profiles reveal candidate metabolic pathways linked to oocyte competence (17). These observations support a distinct local follicular-dominant evidence category while also showing why clinical association, molecular discovery, and mechanistic inference need to be interpreted separately.

#### 2.4.2 Implantation, live birth, and maternal-neonatal outcomes

Early laboratory outcomes do not capture the complete effect of a phenotype. Lean-obese comparisons, insulin-resistance cohorts, dyslipidemia analyses, and transfer studies show that associations can differ across embryo quality, clinical pregnancy, live birth, cumulative live birth, and pregnancy complications (3, 12, 14, 16). A marker may predict an intermediate endpoint without improving cumulative live birth, while a maternal metabolic phenotype may become more relevant after transfer. Outcome definitions, denominators, freeze-all strategies, and duration of follow-up must therefore be reported alongside the phenotype itself.

This outcome-layer approach also limits overinterpretation. Associations between maternal PCOS and pregnancy or neonatal complications in meta-analysis are clinically important, but between-study heterogeneity and incomplete phenotype stratification restrict causal attribution (13). The appropriate conclusion is not that all women with PCOS share one elevated ART risk profile, but that metabolic and inflammatory features may identify subgroups requiring different monitoring priorities. Whether those subgroups benefit from different treatment remains a separate intervention question.

### 2.5 Working phenotypes and a testable compartmental susceptibility model

For evidence synthesis, findings can be grouped into five overlapping working patterns (**Table 2**): metabolic-dominant, inflammation/redox-dominant, local follicular-dominant, endometrial/immune-maternal, and mixed. The categories intentionally overlap because traits are correlated and may change across treatment stages. Their purpose is to define reproducible axes for analysis, not to assign a patient to a clinical subtype.

**Table 2:** Working metabolic-inflammatory phenotypes for evidence synthesis in ART.

Working phenotype (analytic only)	Representative measures	Dominant compartment	Observed associations/hypothesis-generating relevance	Main evidence boundary
Metabolic-dominant	BMI/adiposity; fasting or post-load glucose and insulin; HOMA-IR; lipids; TyG-BMI	Systemic, with possible follicular correlates	Reported associations with ovarian response, embryo development and some transfer/pregnancy outcomes; not treatment-defining	Correlated exposures and residual confounding; cutoffs are assay- and population-dependent
Inflammation/redox-dominant	Leukocyte or inflammatory indices; cytokines; oxidative-stress and antioxidant markers	Systemic and follicular	Exploratory associations with oocyte/embryo and implantation-related outcomes	Nonspecific and time-sensitive markers; causal mediation is unproven
Local follicular-dominant	Follicular-fluid metabolites/cytokines; small extracellular vesicles; granulosa-cell expression; immune profiles; omics	Follicular	Discovery associations with oocyte/embryo measures; incremental clinical value remains unproven	Late or invasive sampling; small cohorts; multiplicity, platform and batch effects
Endometrial/immune-maternal	uNK cells; macrophage/dendritic/T-cell profiles; IL-15, IL-18, IL-6, IL-8; receptivity markers; maternal metabolic variables	Endometrial, maternal, and pregnancy	Tissue-level associations with receptivity and pregnancy biology; no validated treatment threshold	Cycle-phase, hormonal preparation and phenotype dependence; no validated immune threshold for treatment selection
Mixed	Abnormalities spanning multiple domains or compartments	Multicompartment	Hypothesis: cross-compartment burden or discordance may improve stage-specific risk stratification	Not a validated subtype; requires reproducible clustering, external validation and interaction testing

These categories overlap and are intended only for evidence synthesis. The fourth column describes reported associations or testable hypotheses, not treatment indications. No validated threshold allows these categories to be used as diagnostic subtypes or treatment algorithms. Genetic and epigenetic determinants are cross-cutting modifiers rather than a separate phenotype class.

These working phenotypes are analytic labels, not diagnostic classifications or treatment algorithms. Cluster analysis has identified a metabolically disturbed PCOS subgroup associated with adverse reproductive outcomes, but cluster membership depends on variable selection, scaling, population, and endpoint and requires external replication (18). Within the compartmental susceptibility model, the key test is not whether a cluster has a poorer prognosis, but whether paired systemic-local information adds reproducible predictive value beyond age, ovarian reserve, prior response, and protocol, and whether a prespecified phenotype-by-treatment interaction identifies a treatment that improves cumulative live birth or safety. The model is therefore explicitly hypothesis-generating.

### 2.6 Genetic and epigenetic determinants of phenotype

PCOS susceptibility is strongly polygenic. A large genome-wide meta-analysis showed that the overall genetic architecture was broadly shared across commonly used diagnostic criteria, arguing against a simple one-gene/one-phenotype model (19). More recently, a multi-ancestry analysis identified 94 independent susceptibility loci, including 73 previously unreported loci, and prioritized granulosa-cell biology, AMH-related regulation, and PPARG-linked metabolic pathways (20). These findings support heterogeneity across reproductive and metabolic axes but do not define ART-ready subtypes.

Genetic background is therefore best treated as a cross-cutting modifier of the working phenotypes rather than as a sixth clinical category. At present, polygenic scores have not been prospectively validated to select gonadotropin dose, trigger strategy, embryo-transfer preparation, or immune/metabolic adjuncts in women with PCOS. Any future genetic stratifier will need to add information beyond age, ovarian reserve, BMI, previous ovarian response, and treatment protocol, and its performance should be evaluated across ancestries before clinical use.

Epigenetic changes may link inherited susceptibility to endocrine and metabolic exposures, but their direction of causality is especially difficult to establish. In PCOS endometrium, altered expression of HOXA10, GAB1, and SLC2A4 together with higher HOXA10 promoter methylation has been reported; a small intervention subset showed changes after metformin plus a carbohydrate-controlled diet (21). Because methylation and transcription are tissue-, cell-composition-, cycle-phase-, medication-, and metabolic-state-dependent, these signals are better regarded as potentially modifiable biological readouts than as fixed determinants of ART outcome.

## 3 Follicular-oocyte pathway: metabolic and immune crosstalk

### 3.1 Follicular metabolism, redox balance, and local signaling

The ovarian follicle is where systemic metabolic exposures meet the developing oocyte. Follicular fluid is not simply a passive ultrafiltrate of plasma; together with cumulus and mural granulosa cells, it forms a metabolically active niche in which endocrine signals, nutrients, lipids, redox balance, cytokines, and cellular metabolites are integrated during follicular growth. In PCOS, this niche is exposed to different combinations of hyperinsulinemia, adiposity, dyslipidemia, androgen excess, and low-grade inflammation. The local state may therefore vary considerably among patients with the same diagnosis and may not be captured by BMI or a fasting insulin index alone.

Clinical cohort evidence supports associations between systemic metabolic dysfunction and early IVF/ICSI outcomes. In women with PCOS, glucose-related indices, adiposity, lipid measures, and inflammatory indices have been linked to oocyte retrieval, metaphase-II oocytes, fertilization, and embryo development (7, 8). These retrospective mediation models should not be read as evidence that adipose-derived inflammation directly damages the embryo. Age, ovarian reserve, infertility duration, gonadotropin dose and protocol, trigger choice, laboratory practice, and the number of available oocytes can confound exposure-mediator, mediator-outcome, and exposure-outcome relations. The studies are therefore treated here as hypothesis-generating observational evidence.

Local molecular studies add mechanistic resolution but not equivalent clinical certainty. Altered ANGPTL4 expression in granulosa cells, proteomic differences in follicular-fluid-derived small extracellular vesicles, and integrated follicular multi-omics identify candidate pathways or markers associated with PCOS or oocyte competence (9, 10, 17). Their small or selected samples, late-cycle sampling, multiplicity, platform effects, and lack of external validation mean that they cannot establish a systemic-to-local causal sequence or support treatment selection.

Oocyte maturation requires coordinated energy production and metabolite exchange between the oocyte and its surrounding cumulus cells. Glucose utilization, lipid turnover, amino-acid availability, and mitochondrial function therefore provide plausible routes through which metabolic dysfunction can affect competence. In PCOS cohorts, correlated glucose and lipid abnormalities make it difficult to assign the observed reproductive association to a single substrate. This is particularly important when statistical models include several biologically connected markers: attenuation after adjustment or mediation analysis can indicate shared information, but it does not identify the direction of molecular traffic within the follicle (8).

Redox status is another potential integrator. A disturbed balance between reactive species and antioxidant defenses has been documented in the follicular environment of women with PCOS and has been associated with abnormal folliculogenesis and reproductive outcomes (22). Oxidative stress may interact with lipid peroxidation, mitochondrial function, and inflammatory signaling rather than acting as an isolated pathway. The available human evidence, however, is sensitive to the selected assay, timing of sampling, follicle pooling, and whether the analysis concerns serum, follicular fluid, cumulus cells, or the oocyte itself. A redox marker measured at retrieval may reflect both the pre-existing phenotype and the preceding stimulation cycle.

Culture conditions can further modify cellular metabolism. Exploratory work on physiological oxygen tension during human cumulus-oocyte-complex maturation illustrates that oxygen exposure influences metabolic behavior *in vitro* (23). This evidence is useful for understanding the sensitivity of the oocyte-cumulus unit, but it should not be generalized directly to conventional IVF outcomes in all women with PCOS. Likewise, *in vitro* maturation offers an important management context for women at high risk of excessive response, yet evidence concerning laboratory maturation, embryo competence, cumulative live birth, and safety must be considered separately (24, 25). The metabolic vulnerability of the follicle creates a rationale for optimized culture and maturation strategies; it does not by itself establish superiority over conventional treatment.

### 3.2 Immune composition and intercellular communication

Inflammatory indices have been associated with adiposity, lipid metabolism, and embryo development in retrospective IVF/ICSI cohorts, including statistical mediation of selected associations (7). Serum inflammatory factors also show outcome associations that differ across BMI strata (26). These analyses are compatible with metabolic-inflammatory interdependence, but residual confounding, reverse directionality, treatment selection, and correlated exposures preclude causal language. We therefore use them to generate candidate pathways, not to infer that inflammation is a proven mediator of embryonic impairment.

The local immune compartment is more complex than a circulating inflammatory score. High-dimensional profiling has identified distinct immune-cell and cytokine signatures in follicular fluid and peripheral blood, including PCOS-associated differences within the follicular compartment (11). This compartmental divergence matters for biomarker development: a blood marker may be convenient yet biologically remote, whereas follicular sampling is local but available only after treatment has begun. Small extracellular vesicles provide another potential communication route. Their protein cargo may integrate metabolic and inflammatory information, but isolation methods, normalization, follicle pooling, and analytical batch effects currently limit comparability (10).

The most defensible interpretation is a network of candidate interactions rather than a linear causal chain. Metabolic substrates, granulosa-cell responses, immune cells, cytokines, redox pathways, and extracellular vesicles may interact within the peri-oocyte environment, but current human studies identify possible nodes rather than a completed mechanism. Stronger inference will require prospective paired sampling, standardized assays, explicit control of stimulation-related confounding, and experimental or intervention-based validation.

### 3.3 Oocyte yield, embryo competence, and cumulative outcome

Ovarian response and developmental competence should not be conflated. Women with PCOS may produce many oocytes while still showing heterogeneity in maturity, fertilization, embryo development, OHSS risk, and cumulative live birth. Analyses of retrieved-oocyte number suggest a nonlinear balance between the opportunity to obtain transferable embryos and the safety or efficiency costs of a very high response (27). Follicular output rate similarly captures response relative to the available follicle pool and has been associated with cumulative pregnancy and live-birth outcomes in PCOS subgroups (28). Neither metric directly measures intrinsic oocyte quality, and both depend on protocol and denominator definitions.

Embryo morphology and blastulation are useful intermediate outcomes, but they remain downstream of oocyte number and laboratory practice. Their interpretation should therefore distinguish per-oocyte, per-embryo, per-transfer, and cumulative-cycle denominators. A metabolic phenotype that is associated with fewer high-grade embryos may still have an uncertain effect on cumulative live birth if more oocytes are retrieved or if freeze-all and repeated transfers compensate. Conversely, similar embryo morphology does not exclude a maternal metabolic contribution to miscarriage or obstetric complications.

Taken together, clinical and molecular evidence supports compartment-specific heterogeneity across follicular metabolism, immune activity, and redox balance. High oocyte yield and apparently favorable embryo morphology do not exhaust the possible effect of the phenotype; conditions at transfer and during pregnancy must be considered separately.

## 4 Endometrial-maternal pathway: receptivity, implantation, and pregnancy

### 4.1 Receptivity is more than endometrial thickness

The outcome of ART cannot be assigned to the embryo alone. After fertilization and culture, implantation depends on embryo competence, endometrial development, transfer timing, and the maternal metabolic and immune environment. PCOS studies often measure these components incompletely, making pathway attribution difficult. Evidence from systemic metabolic indices in frozen embryo transfer, embryo-focused blastulation prediction, and meta-analytic maternal-neonatal outcomes demonstrates that apparently similar clinical endpoints can arise from different stages of the ART pathway (12, 13, 29).

Endometrial thickness is routinely available but is an incomplete proxy for receptivity. In women with PCOS, reduced thickness has been associated with selected neonatal complications, yet retrospective associations do not establish that thickness itself is the causal exposure (30). Hormonal environment, vascular function, embryo characteristics, transfer protocol, and unmeasured metabolic traits may contribute. Thickness should therefore be interpreted as one component of transfer assessment, not a standalone explanation for implantation or neonatal risk.

### 4.2 Endometrial immune profile and metabolic context

The persistence of phenotype associations in frozen embryo transfer provides indirect evidence that preconception metabolism may remain relevant after the stimulation cycle. A composite triglyceride-glucose-BMI measure has been associated with miscarriage, live birth, and selected obstetric outcomes among women with PCOS undergoing FET (12). Insulin resistance and beta-cell dysfunction have likewise been linked to pregnancy loss, live birth, or macrosomia in retrospective transfer cohorts (16, 31). These associations may reflect systemic maternal physiology, endometrial function, placentation, correlated adiposity, treatment selection, or residual confounding; they do not, by themselves, identify an endometrial mechanism.

Direct tissue studies indicate that the endometrial immune profile is not a simple mirror of circulating inflammation. In late-secretory endometrium from infertile women with PCOS, Matteo et al. reported lower proportions of CD56+/CD16− and CD56bright/CD16− uterine natural killer (uNK) cells together with reduced expression of IL-15, IL-18, and CXCL10, although the control group was small (32). In proliferative-phase endometrium, IL-18 expression was higher in PCOS overall and was particularly increased in overweight women, while the difference was not significant in normal-weight women (33). The apparently divergent findings underscore the importance of cycle phase, adiposity, assay platform, and the distinction between immune-cell abundance and immune function.

A larger observational study of 100 women with PCOS and 95 fertile controls found higher endometrial macrophage, dendritic-cell, and CD8+ T-cell measures in normoweight PCOS, with associations between CD56+ NK/CD163+ M2 macrophage measures and insulin sensitivity (34). A phenotype-stratified endometrial study further linked insulin resistance, hyperandrogenemia, and obesity with changes in IL-15-related signaling, IL-6/IL-8, uNK-cell profiles, and receptivity-related molecules (35). These data support immunometabolic coupling at the tissue level, but they remain cross-sectional or observational and do not establish that immune-cell counts or cytokine concentrations should direct FET preparation or immunomodulatory treatment.

Endometrial transcriptomics adds another layer. During the implantation window, a small prospective RNA-sequencing study comparing infertile obese women with PCOS and normal-weight infertile controls identified differences in inflammatory, insulin-signaling, fatty-acid-metabolism, and cellular-stress pathways (36). The study is mechanistically informative but illustrates the inferential limits of high-dimensional tissue data: sample size was small, obesity and PCOS were difficult to separate completely, and pathway-level signals were not linked to a validated treatment decision or cumulative live birth.

Taken together, uNK cells and cytokines are promising components of an endometrial phenotype, not stand-alone clinical biomarkers. Cell number, activation state, tissue localization, cycle timing, ovulatory status, obesity, insulin resistance, and hormonal preparation can all alter interpretation. No reproducible uNK- or cytokine-based threshold has been validated to select transfer regimen or adjunctive immune therapy in PCOS. Studies intended to move this field toward ART decision-making should align sampling with the implantation window, use prespecified immune panels, and relate tissue findings to embryo-independent endpoints, live birth, and safety.

### 4.3 Transfer preparation and the endometrial microenvironment

Transfer-cycle strategy is a potential effect modifier. Retrospective comparison of mildly stimulated and artificial FET cycles in overweight or obese women with PCOS suggests that preparation method may be associated with pregnancy outcomes, while a more recent comparative trial of letrozole-stimulated and hormone-replacement cycles addresses the same clinical decision from a different design perspective (37, 38). Protocol definitions, ovulatory status, luteal support, cancellation, and clinician selection must be retained when comparing these studies. Evidence for one preparation strategy should not be generalized to every metabolic-inflammatory phenotype.

The gut and lower-genital-tract microbiomes are plausible upstream modifiers of metabolic inflammation, but they are not validated ART decision variables. A recent review summarizes gut-microbiota biodiversity and metabolic intervention in PCOS (6). In a randomized double-blind trial of women with PCOS undergoing IVF, vitamin D plus probiotic supplementation improved insulin-related and inflammatory markers but did not establish a live-birth benefit (39). These data support mechanistic interest, not routine microbiome-directed treatment during IVF.

### 4.4 Clinical outcomes and registry-scale context

Pregnancy is a sequence of outcomes, not a single endpoint. Early miscarriage, later pregnancy loss, hypertensive disorders, gestational metabolic complications, preterm birth, birth weight, and neonatal morbidity may have different determinants. Cohort evidence associates insulin resistance with early miscarriage and macrosomia (16). Meta-analysis of ART pregnancies links maternal PCOS with several pregnancy and neonatal complications, although heterogeneity in PCOS definitions, BMI adjustment, embryo-transfer strategy, plurality, and outcome ascertainment limits causal interpretation (13).

Large ART registries provide an important external frame for these phenotype-focused studies. A SART CORS analysis of linked autologous cycles included 447,042 patients and more than one million cycles from the 2014–2020 era and demonstrated that cumulative live-birth patterns vary materially by age, treatment era, and infertility diagnosis (40). In Europe, the ESHRE registry report for 2020 compiled 923,318 ART cycles from 1,440 clinics in 41 countries, illustrating both the scale of contemporary ART and the heterogeneity of national reporting and treatment practice (41).

These registries strengthen denominator discipline, benchmarking, and external validation, but they should not be overread as proof of a PCOS metabolic-inflammatory mechanism. Routine registry variables generally do not capture detailed glucose-insulin phenotyping, endometrial uNK/cytokine profiles, follicular immune states, or local multi-omics. Their greatest value for precision ART is therefore complementary: registry-scale outcomes can test whether a prediction model or proposed subgroup generalizes, whereas mechanistic cohorts are needed to define the biological phenotype in the first place.

The endometrial-maternal pathway therefore extends metabolic-inflammatory phenotyping beyond the embryology laboratory. Its clinical relevance is clear, but mechanistic attribution remains less direct than in the follicular compartment. Any biomarker or prediction model intended for this stage should specify the event, sampling window, comparator, denominator, and clinical action it is intended to inform.

## 5 Biomarkers, multi-omics, and prediction models for phenotype-guided ART

### 5.1 Biomarkers must improve a decision

A candidate biomarker becomes clinically useful only if it improves a defined decision beyond information already available from age, ovarian reserve, prior response, BMI, and protocol. Systemic markers are accessible but nonspecific; follicular metabolites, granulosa-cell expression, extracellular-vesicle proteins, immune profiles, and metabolomic signatures are biologically closer to the oocyte but are usually measured late in the cycle. Current evidence from follicular-vesicle proteomics, immune profiling, VIP, and blastulation models is predominantly discovery or internally validated evidence (10, 11, 29, 42). Biological novelty should not be equated with clinical utility.

Systemic markers have advantages of low burden, repeatability, and availability before treatment. Composite measures such as TyG-BMI may summarize correlated metabolic dimensions and have shown associations with FET outcomes (12). Yet composites can conceal which component drives prediction, and cutoffs derived from one center may not generalize across ethnic groups, assays, or treatment protocols. AMH is valuable for ovarian-response assessment, but its relationship with miscarriage or pregnancy outcome is not uniform in PCOS. Inflammatory and oxidative-stress measures face similar problems of assay timing, nonspecificity, and confounding.

Local markers may be valuable for mechanistic research, but their current clinical role should be stated conservatively. Follicular-fluid extracellular-vesicle proteomics, multi-omics profiles, immune signatures, and VIP require replication with prespecified assays, blinded outcome assessment, and external validation (10, 11, 17, 42). Because sampling generally occurs at retrieval, these measurements cannot guide the initial stimulation cycle. At present there is insufficient evidence to use them for routine embryo selection, transfer planning, or future-cycle treatment selection.

### 5.2 Prediction models and decision points

Prediction targets in PCOS ART include low or excessive ovarian response, trigger failure, OHSS, oocyte maturity, blastulation, implantation, miscarriage, live birth, and obstetric complications. A model optimized for one target cannot be assumed to perform for another. A blastulation nomogram may help estimate laboratory development but does not directly predict cumulative live birth (29). A pre-trigger OHSS score is positioned later in the cycle and addresses safety rather than efficacy (43). The predictor window and intended action should therefore be part of the model definition.

Clinical usefulness also requires more than discrimination. Studies should report calibration, missing-data handling, optimism correction, internal and external validation, and performance across phenotype and protocol subgroups. Decision-curve analysis can estimate whether acting on a model offers net benefit at plausible thresholds, but it does not replace prospective impact evaluation. Continuous predictors should generally be retained during model development rather than dichotomized at data-derived cutoffs. Updating or recalibration may be preferable to direct transfer when baseline risk differs.

### 5.3 Multi-omics: batch effects, overfitting, and reproducibility

Multi-omics can reveal pathways that routine metabolic testing misses, but the typical high-dimensional, low-sample-size design makes false discovery and overfitting a central concern. Follicular and endometrial studies already illustrate this tension (17, 36, 44). Batch effects can arise from site, recruitment period, specimen handling, storage duration, extraction kit, plate, library preparation, sequencing run, instrument, or bioinformatic preprocessing. If a batch variable is correlated with phenotype or outcome, technical structure can mimic biology; *post hoc* correction cannot reliably rescue a design in which batch and outcome are confounded.

Analytical leakage is equally important. Feature selection, normalization, imputation, dimensionality reduction, or batch correction performed before data splitting can allow information from the validation set to influence model development and inflate apparent performance. Robust studies should prespecify preprocessing and primary features where possible, control multiplicity, use nested resampling for tuning, keep all outcome-informed feature selection inside the training data, and then evaluate a locked pipeline in an independent cohort. Multi-center validation should preserve site and platform structure rather than randomly mixing near-duplicate technical environments.

A molecular signature should also be compared with a parsimonious clinical baseline. Incremental discrimination alone is insufficient: calibration, missing-data handling, transportability across BMI strata and diagnostic criteria, and decision-curve or impact analysis are needed before a test can change stimulation, trigger, transfer, or pregnancy monitoring. Until these standards are met, local molecular panels and current nomograms should be described as discovery or internally validated tools, not implementation-ready instruments.

## 6 Precision ART management according to metabolic-inflammatory phenotype

### 6.1 Pretreatment: risk modification without delaying effective care

Pretreatment is an opportunity to evaluate weight trajectory, glucose tolerance, insulin resistance, blood pressure, lipids, and relevant endocrine comorbidity in line with broader PCOS care principles (2). Lifestyle and metabolic management may be appropriate for long-term health, but any ART-specific benefit should be judged against time to pregnancy, live birth, cumulative live birth, adverse effects, and the cost of delaying treatment. The balance is likely to differ by age, ovarian reserve, severity of metabolic disease, and previous ART history.

Metformin is the clearest example of a context-specific adjunct rather than a phenotype-wide ART therapy. The 2023 international guideline states that adjunct metformin can be considered before and/or during FSH stimulation when a GnRH-agonist long protocol is used, primarily to reduce OHSS risk; the same guideline emphasizes the flexibility of a GnRH-antagonist protocol with agonist trigger and freeze-all for OHSS prevention (2). A Cochrane review found no clear overall live-birth benefit and rated the evidence for major IVF outcomes as low or very low certainty (45). Insulin resistance may strengthen biological plausibility, but no HOMA-IR threshold has been validated to mandate metformin in every IVF cycle.

### 6.2 Stimulation, dose, trigger, and OHSS prevention

PCOS is associated with a tendency toward high ovarian response, but actual response ranges widely. Protocol selection should be anchored in ovarian reserve, age, previous response, and dynamic monitoring; metabolic-inflammatory information may add context but has not been validated as a standalone dosing rule. The same caution applies across pretreatment, stimulation, trigger, transfer preparation, and adjunctive care.

Comparisons of mild and conventional stimulation show that response category and desired endpoint influence the tradeoff between medication burden, oocyte yield, cancellation, and cumulative success (46). PCOS-specific retrospective comparisons of gonadotropin preparations and studies of progestin-primed stimulation add practical evidence, but protocol selection and confounding remain concerns (47, 48). A nonlinear association between total gonadotropin dose and cumulative live birth in advanced-age women with PCOS cautions against assuming that more medication produces proportional benefit (49). The reported threshold is cohort-specific and requires external validation before clinical use.

OHSS prevention is a safety-critical decision. A pre-trigger risk score illustrates how information available during stimulation can be combined to estimate moderate-to-severe OHSS risk (43). Trigger choice and a freeze-all strategy can then be considered in the context of response, luteal support, and the planned transfer pathway. Comparative data on human chorionic gonadotropin (hCG) and gonadotropin-releasing hormone (GnRH)-agonist triggers in PCOS contribute to this decision, but retrospective selection and subsequent FET strategy remain important sources of bias (50).

### 6.3 Embryo/FET strategy and adjunctive therapies

Embryo strategy should distinguish immediate transfer success from cumulative-cycle outcome. Oocyte number, blastulation probability, embryo stage, cryopreservation, and transfer day all affect the available treatment sequence. Retrospective work comparing transfer days in PCOS provides useful hypothesis-generating evidence but remains sensitive to embryo selection and transfer policy (51). A freeze-all approach may reduce immediate OHSS-related risk in selected high responders, yet its downstream effectiveness depends on embryo survival, FET preparation, and cumulative follow-up.

Endometrial preparation should likewise be linked to ovulatory function, metabolic phenotype, convenience, safety, and evidence quality. Studies comparing stimulated and artificial FET cycles in PCOS suggest that cycle regimen may matter (37, 38), but evidence is not sufficient to prescribe a regimen solely from BMI or insulin-resistance status. Prospective phenotype-stratified comparisons should test interactions rather than infer them from subgroup significance.

Adjunctive interventions should not be discussed as one evidentiary category. For inositol, a 2025 meta-analysis of intervention studies reported higher clinical pregnancy and top-grade embryo rates, but live-birth evidence was imprecise and several ovarian outcomes were highly heterogeneous (52). For antioxidant therapy, a 2025 meta-analysis of astaxanthin trials found improvements in selected antioxidant and embryo-related measures but no consistent pregnancy benefit (53), while a double-blind L-carnitine trial did not improve IVF/ICSI outcomes (54). A 2026 meta-analysis of 22 acupuncture RCTs reported higher clinical pregnancy and live-birth rates, but certainty ranged from very low to moderate and intervention/stimulation protocols were heterogeneous (55). These signals justify further trials; they do not establish a metabolic-inflammatory phenotype in which any of these adjuncts should routinely be prescribed.

Evidence is weaker for microbiome-directed supplements and anticoagulation. The small probiotic/vitamin D IVF trial primarily demonstrated metabolic and inflammatory biomarker changes rather than a proven improvement in cumulative live birth (39). For low-molecular-weight heparin (LMWH), we removed the previously cited protocol-only study and do not use an unpublished trial design as efficacy evidence. ESHRE good-practice recommendations for recurrent implantation failure do not recommend LMWH to increase pregnancy or live birth in women with recurrent implantation failure without a specific thrombotic indication (56). There is no PCOS-specific evidence that supports routine LMWH solely on the basis of a metabolic-inflammatory phenotype. Thus, among the adjuncts considered here, only metformin has a guideline-defined IVF scenario, and even that recommendation is protocol-dependent.

Clinical precision is therefore better conceived as a sequence of decisions than as a single phenotype-based protocol. A biomarker is useful only if it changes one of those decisions and improves a meaningful outcome; prognostic separation alone is insufficient evidence of treatment benefit.

## 7 ART outcomes and translational integration

### 7.1 Outcome hierarchy and denominator discipline

An interpretable outcome hierarchy separates laboratory surrogates, cycle-level reproductive outcomes, cumulative effectiveness, safety events, and maternal-neonatal outcomes. Retrieved oocytes, mature-oocyte rate, fertilization, embryo grade, and blastulation describe biological and laboratory performance. Clinical and ongoing pregnancy are closer to patient benefit but do not capture pregnancy loss. Live birth and cumulative live birth are therefore the preferred effectiveness endpoints, while OHSS, obstetric complications, and neonatal outcomes remain essential safety endpoints.

Denominators determine interpretation. Per-retrieval and per-transfer rates answer different questions, and cumulative live birth requires a defined follow-up window and accounting for all embryos from one stimulation. Oocyte-number and follicular-output studies illustrate why a favorable laboratory response may have a nonlinear relationship with cumulative outcome (27, 28). Blastulation models address a narrower target (29), whereas dyslipidemia and gonadotropin-dose studies evaluate complete-cycle outcomes (3, 49). Results should not be compared without aligning these units of analysis.

### 7.2 From prognosis to clinically actionable evidence

Across the evidence base, metabolic-inflammatory features are associated with different endpoints at different stages. Adiposity and glucose-lipid-inflammatory measures are associated with oocyte and embryo outcomes (7, 8); lean-obese comparisons show prognostic heterogeneity (14, 15); and insulin-resistance or beta-cell-function measures are associated with miscarriage and live birth in transfer cohorts (16, 31). Cluster analysis also identifies groups with different reproductive outcomes (18). Under the evidence hierarchy used here, these data support association or risk stratification—not causal mediation or treatment selection.

These findings support risk stratification, but they do not yet demonstrate treatment effect modification. A subgroup with poorer prognosis does not necessarily benefit more from an intervention. To justify phenotype-guided care, studies must compare alternative management strategies within prespecified phenotypes and test interaction on clinically meaningful outcomes. External validation of phenotype definitions should precede, or be embedded within, such trials.

OHSS is the most immediate ART-specific safety concern, and prediction near triggering may permit actionable prevention (43). Safety assessment should then continue through pregnancy. Meta-analytic evidence indicates associations between maternal PCOS after ART and several obstetric or neonatal outcomes (13). Cohort evidence linking metabolic-inflammatory status, insulin resistance, BMI, and endometrial thickness to pregnancy or neonatal complications suggests that risk is not uniform (16, 30, 57, 58).

The clinical aim is not to label all pregnancies after PCOS ART as high risk. It is to determine whether an ART-relevant phenotype adds calibrated risk information beyond age, BMI, plurality, embryo-transfer strategy, pre-existing disease, and established obstetric predictors. Studies should report absolute as well as relative risk and avoid conditioning only on women who achieved pregnancy when estimating the entire ART pathway.

### 7.3 A translational decision sequence

The evidence can be organized into five decision points. At initial assessment, routine metabolic and reproductive variables identify general health needs and establish baseline risk. Before stimulation, ovarian reserve, prior response, and metabolic phenotype inform the expected response range. During stimulation, dynamic follicular and hormonal data refine dose and OHSS estimates. After retrieval, embryo and local follicular information may update prognosis for transfer or later cycles. During transfer and pregnancy, maternal metabolic and endometrial factors shift attention toward implantation, pregnancy maintenance, and obstetric surveillance.

Current evidence is most mature for routinely available clinical variables and dynamic ovarian-response measures, with the clearest actionable evidence centered on established OHSS-prevention strategies. Follicular omics, extracellular-vesicle signatures, endometrial immune profiles, and most multivariable models remain discovery or early validation tools. The near-term value of the framework is therefore to improve study design, evidence weighting, and risk communication rather than to create a phenotype-based treatment menu.

## 8 Discussion, limitations, and future directions

### 8.1 Integrated interpretation and current controversies

The evidence is most coherent when follicular and endometrial pathways are treated as related but non-interchangeable. Clinical cohorts associate glucose, lipid, adiposity, and inflammatory measures with oogenesis and embryogenesis (7, 8), while local studies identify granulosa-cell, extracellular-vesicle, immune, and multi-omics signals that are not captured by BMI alone (9–11, 17). After embryo creation, transfer cohorts and tissue studies associate metabolic state, uNK/cytokine networks, and endometrial transcription with implantation or pregnancy-related phenotypes (12, 16, 32, 34–36). The appropriate level of inference is association and biological plausibility; the present evidence does not demonstrate a single causal pathway.

The principal conceptual contribution of this review is the compartmental susceptibility hypothesis, not the five working phenotype labels themselves. The model predicts that the clinical meaning of a systemic metabolic-inflammatory trait depends on whether a stage-specific local compartment is susceptible, and that cross-compartment concordance or discordance may be more informative than either measurement alone. This proposition can be rejected if paired systemic-local measurements fail to add reproducible information beyond standard clinical predictors, or if phenotype-by-treatment interactions do not improve patient-important outcomes. In this way, the framework is intended to generate prospective tests rather than to repackage existing mechanisms as a new classification.

Causal interpretation is particularly vulnerable in retrospective mediation analyses. Age, ovarian reserve, infertility duration, baseline BMI and weight change, metabolic treatment, stimulation protocol and total gonadotropin exposure, trigger strategy, laboratory practice, fertilization method, embryo stage and quality, freeze-all policy, FET preparation, luteal support, and the number of embryos transferred can influence both intermediate variables and ART outcomes. Conditioning on retrieval, transfer, or achieved pregnancy can introduce additional selection bias. Consequently, an apparent indirect effect through inflammation or lipids may reflect residual confounding, model specification, or treatment pathways rather than a biological mediator.

### 8.2 Limitations of the evidence base and this review

The evidence base is dominated by retrospective and frequently single-center studies, including many ART outcome, transfer, and prediction analyses (12, 14, 16, 29, 31, 37, 43, 47, 49). We therefore considered six recurrent bias domains when weighting findings (1): baseline patient selection and confounding by age, ovarian reserve, infertility duration, BMI trajectory and metabolic comorbidity (2); stimulation-related confounding by protocol, starting and total gonadotropin dose, trigger and ovarian response (3); embryology-related variation in laboratory practice, insemination method, embryo stage/quality and cryopreservation (4); transfer-related variation in fresh versus freeze-all strategy, FET preparation, luteal support and number of embryos transferred (5); post-transfer selection, plurality and obstetric comorbidity; and (6) analytic bias from missing data, multiple testing, data-driven cutoffs or internal-only validation. These domains are summarized in the evidence hierarchy in **Table 1** and constrain the strength of every clinical inference.

Mechanistic studies add biological resolution but not necessarily stronger clinical inference. Follicular-fluid, immune, transcriptomic, and metabolomic studies often rely on small samples, invasive or late-cycle sampling, platform-specific preprocessing, multiple testing, and limited external replication (10, 11, 17, 36, 44). Endometrial immune studies additionally vary by cycle phase, ovulatory status, BMI, hormonal preparation, and cell panel (32–35). Prediction studies frequently emphasize discrimination while reporting less on calibration, external validation, or clinical impact (29, 43). These limitations are why candidate biomarkers are assigned discovery-level weight unless replicated and prospectively validated.

This article remains a narrative, hypothesis-driven review rather than a *de novo* systematic review or meta-analysis; no pooled effect estimates or formal study-by-study risk-of-bias instrument was applied. To address the risk of treating unlike evidence as equivalent, we added an explicit evidence-weighting framework and qualitative bias domains (**Table 1**), removed protocol-only studies from supporting claims, and revised causal language throughout. Study selection was guided by directness to the follicular-oocyte and endometrial-maternal questions, recency, biological coherence, and relevance to ART decisions. Because the review did not use a prespecified systematic-search protocol, relevant reports may still have been missed.

### 8.3 Priorities for research and clinical translation

Phenotype measurement needs greater standardization. Studies should report diagnostic criteria, continuous metabolic variables, assay methods, sampling time, medication exposure, cycle phase, recent weight change, stimulation and trigger protocol, embryo-transfer strategy, and a prespecified outcome hierarchy. Paired systemic, follicular, and endometrial sampling would provide the most direct test of the compartmental susceptibility hypothesis and determine when a blood measure approximates—or fails to approximate—a local reproductive compartment (2, 11, 34, 35). Genetic ancestry and epigenetic measurements should be incorporated where biologically justified, with explicit attention to tissue specificity and confounding (19–21).

Prediction research needs an equally explicit validation pathway. Multi-center datasets should support external validation across laboratories, protocols, ancestries, and lean and obese phenotypes; calibration, clinical utility, missing-data handling, and model updating should accompany discrimination (29, 43). Multi-omics signatures should be analytically locked before external testing and compared against parsimonious clinical baselines (17, 44). Linkage to large outcome registries such as SART CORS or national/European reporting systems could provide a pragmatic route to transportability testing, provided the phenotype variables and outcome definitions are compatible (40, 41).

The decisive clinical question is treatment-effect modification. Prospective studies should prespecify biologically credible phenotype strata, retain the relevant continuous measures, and compare alternative management strategies using cumulative live birth, OHSS, time to pregnancy, treatment burden, and maternal-neonatal outcomes. A marker that predicts prognosis is not automatically a marker that predicts treatment benefit. Interaction testing should be planned *a priori*, and improvement in a surrogate endpoint should be treated as mechanistic support unless it is linked to a patient-important outcome.

Dynamic phenotyping may ultimately be more useful than a fixed label. Metabolic status before treatment, ovarian response during stimulation, local follicular information at retrieval, endometrial immune state around transfer, and maternal measures during pregnancy represent different decision windows (34, 37, 38, 43). Explainable decision-support systems could update risk across these windows, but they require prospective evaluation of calibration, fairness, clinician use, and unintended consequences.

Implementation should be studied alongside accuracy. Even a statistically strong model may fail if the assay is inaccessible, results arrive after the relevant decision, thresholds do not map to a specific action, or treatment burden outweighs benefit. Feasibility, cost, patient preferences, net benefit, and cross-center reproducibility should therefore be core outcomes of precision-ART research, not afterthoughts.

## 9 Conclusion

Metabolic-inflammatory heterogeneity is relevant to ART in PCOS, but the current evidence supports an evidence-weighted research framework rather than a new clinical subtype system. The proposed compartmental susceptibility model treats systemic metabolic burden as background exposure and asks whether stage-specific follicular or endometrial vulnerability adds reproducible information and modifies treatment response. High-level evidence is strongest for established clinical risk assessment and OHSS-prevention strategies; most local immune, extracellular-vesicle, multi-omics, and prediction signals remain exploratory. Precision ART will require paired systemic-local phenotyping, explicit control of ART-specific confounding, external validation, and prospective tests of phenotype-by-treatment interaction on cumulative live birth and maternal-neonatal safety.
